# Molecular Epidemiology and Assemblage Typing of *Giardia duodenalis* in School-Age Children Situated along the Southern Shoreline of Lake Malawi, Malawi

**DOI:** 10.4269/ajtmh.23-0156

**Published:** 2023-08-07

**Authors:** John Archer, Lucas J. Cunningham, Alexandra Juhàsz, Sam Jones, Ffion Doull, James E. LaCourse, Bright Mainga, Peter Makaula, Sekeleghe A. Kayuni, Janelisa Musaya, J. Russell Stothard

**Affiliations:** ^1^ Department of Tropical Disease Biology, Liverpool School of Tropical Medicine, Liverpool, United Kingdom;; ^2^ Institute of Medical Microbiology, Semmelweis University, Budapest, Hungary;; ^3^ Laboratory Department, Mangochi District Hospital, Mangochi, Malawi;; ^4^ Malawi Liverpool Wellcome Trust Programme of Clinical Tropical Research, Queen Elizabeth Central Hospital, Blantyre, Malawi;; ^5^ Department of Pathology, School of Medicine and Oral Health, Kamuzu University of Health Sciences, Blantyre, Malawi;; ^6^ MASM Medi Clinics Limited, Medical Aid Society of Malawi, Area 12 Medi Clinic and Head Office, Lilongwe, Malawi

## Abstract

Almost all human giardiasis infections are caused by *Giardia duodenalis* assemblages A and B. Differentiation between human infections with these assemblages, as well as between single-assemblage (A or B) and mixed-assemblage (A and B) infections, is therefore needed to better understand the pathological impact of infection with either, or both, assemblages. We assessed the prevalence of *G. duodenalis* assemblages A and B using 305 fecal samples provided by school-age children situated along the southern shoreline of Lake Malawi. Concurrently, intestinal pathology data were also collected to test for association(s) between assemblage infection status and intestinal health. Prevalence of *G. duodenalis* infection was 39.3% by real-time polymerase chain reaction. Of all identified infections, 32% were single *G. duodenalis* assemblage A and 32% were single *G. duodenalis* assemblage B, whereas 33% were mixed-assemblage infections. Fifteen unique *G. duodenalis* assemblage A and 13 unique *G. duodenalis* assemblage B β-giardin haplotypes were identified. There was a positive association between single infection with *G. duodenalis* assemblage B and both self-reporting of abdominal pain (odds ratio [OR]: 3.05, *P =* 0.004) and self-reporting of diarrhea (OR: 3.1, *P =* 0.003). No association between single infection with assemblage A and any form of intestinal pathology was found. Additionally, there was a positive association between mixed-assemblage infections and self-reporting of abdominal pain (OR: 3.1, *P =* 0.002). Our study highlights the importance *G. duodenalis* assemblage typing and reaffirms the need for improved access to water, sanitation and hygiene infrastructure in rural areas of low- and middle-income countries.

## INTRODUCTION

Human giardiasis is a globally important intestinal parasitic disease caused by infection with the eukaryotic protozoan diplomonad *Giardia duodenalis* (syn. *Giardia intestinalis*, *Giardia lamblia*).^1^^–^^3^ This parasite is primarily considered a waterborne pathogen that has a direct, fecal-oral life cycle.^4^ As such, only a definitive host is required for effective transmission.

Although cosmopolitan in distribution, prevalence of human giardiasis can be particularly high in rural areas of low- and middle-income countries (LMICs) where adequate water, sanitation, and hygiene (WASH) infrastructure is lacking, including those in sub-Saharan Africa.^4^^–^^7^ In these areas, human giardiasis predominantly afflicts children ≤ 15 years of age, which can severely impact normal childhood development.^3^^,^^4^^,^^8^ Of the eight known morphologically identical but genetically distinct *G. duodenalis* taxon assemblages A through H, almost all human infections are caused by assemblages A and B,^9^ although these assemblages are zoonoses, and so transmission of human-infecting giardiasis can be exacerbated by nonhuman animal hosts such as ruminants.^10^^–^^14^

Recent evidence suggests that there may be marked differences in the types of and degree of pathology experienced by those infected with either *G. duodenalis* assemblages A and B, as well as between those burdened with single-assemblage (A or B) or mixed-assemblage (A and B) infections.^5^^,^^15^^–^^25^ As such, differentiation between human infections with *G. duodenalis* assemblages A and B as well as between single- and mixed-assemblage infections using molecular characterization and taxon assemblage typing is needed to better understand the pathological impact of infection with either, or both, assemblages and thus also for improved disease surveillance and control.^4^^,^^5^^,^^26^ These data are especially needed in the context of rural areas of LMICs, where the prevalence of both *G. duodenalis* A and B assemblages is often high and where other parasitic and neglected tropical diseases (NTDs) that cause similar intestinal pathologies, such as intestinal schistosomiasis, can be coendemic.^9^^,^^26^^,^^27^

Lake Malawi is one of seven African Great lakes. With an approximate water surface area of 29,600 km,^2^ Lake Malawi occupies approximately one-fifth of Malawi while also bordering Tanzania and Mozambique along its eastern shoreline. The lake’s most southern shoreline, however, borders Mangochi District, one of Malawi’s 28 districts. Owing to insufficient WASH infrastructure, many members of Mangochi’s multiple communities rely on the lake as a source of drinking water, food (via fishing), a place to bathe, a place to tend livestock, and for recreation.^28^ As such, human–water contact with the lake’s shoreline is commonplace, resulting in a high prevalence of waterborne and zoonotic diseases in this area.^29^^–^^32^

Only limited data on the prevalence of human giardiasis in Mangochi district have been reported.^31^^,^^33^ Furthermore, no data on the prevalence of human-infecting *G. duodenalis* assemblages A and B in this area are currently available. We therefore evaluated the prevalence of human giardiasis infection in school-age children attending four primary schools situated in close proximity to Lake Malawi’s southern shoreline using both point-of-contact rapid diagnostic tests (RDTs) as well as a diagnostic 18S real-time polymerase chain reaction (PCR).^34^ We also aimed to genotype all *G. duodenalis* infections to assemblage level to identify the prevalence of differing infection statuses (single *G. duodenalis* assemblage A infection, single *G. duodenalis* assemblage B infection, or mixed *G. duodenalis* assemblages A and B infection). This was done using nested PCR with subsequent Sanger sequencing together with a confirmatory *G. duodenalis* assemblage A- and B-specific duplex real-time PCR assay.^35^^,^^36^ These data were then compared with pathology data obtained via questionnaire and point-of-contact RDTs to test for any association(s) between assemblage infection status and intestinal pathology.

## MATERIALS AND METHODS

### Ethical considerations.

Ethical approval and research authorizations were approved in the United Kingdom by the Liverpool School of Tropical Medicine (LSTM) Research Ethics Committee (application 17-018) and in Malawi by the National Health Sciences Research Committee (1805). Informed, written assent was obtained from the guardians of all child participants enrolled in the study before any data or clinical sample collection. Study participants who tested positive for *G. duodenalis* infection by QUIK CHEK^®^ RDTs (TECHLAB, Blacksburg, VA) were provided with a single dose of tinidazole under the supervision of the study clinician (S. A. K.). For anonymity, all study participants were assigned an individual study-specific identification code and all laboratory analysis was carried out blinded from any other data.

### Study sample sites.

**Figure 1.**
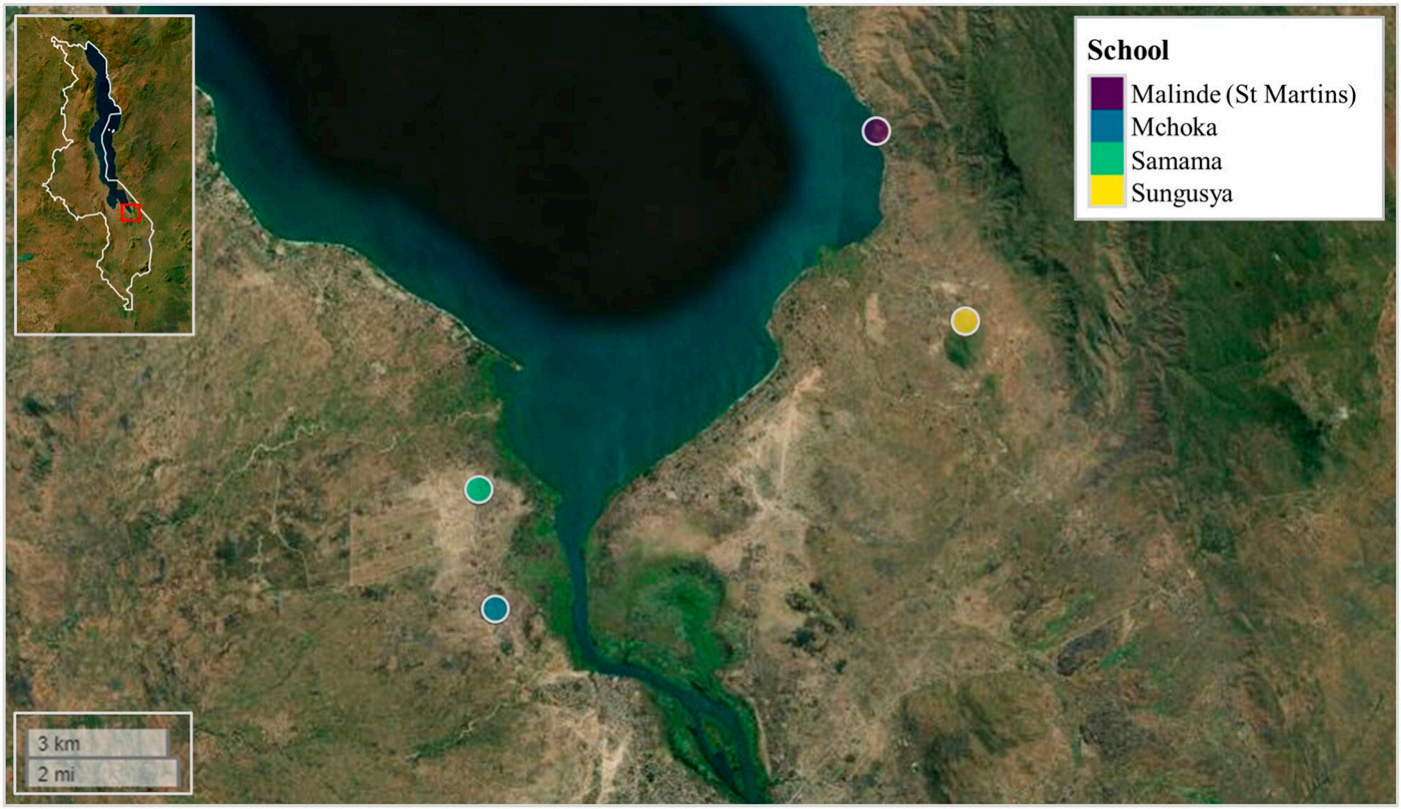
Samama, Mchoka, Sungusya, and Malinde (St. Martin’s) primary schools, situated along the southern shoreline of Lake Malawi, Mangochi District, Malawi. Malawi’s country border can be seen within figure inset (upper left corner). Within figure inset, study area is highlighted in red box. Figure generated using the “map view” package version 2.10.0^37^ within R Studio version 2021.09.0, build 351 (Posit).^38^

Questionnaire responses and fecal material were provided by school-age children situated along the southern shoreline of Lake Malawi, Mangochi district, Malawi, in November of 2021. Four primary schools were selected for parasitological surveillance (two on the western side of the lake and two on the eastern side of the lake) based on their close proximity to the lake’s shoreline and previously identified prevalence’s of urogenital and intestinal schistosomiasis; suggesting frequent community lake water contact.^29^^,^^30^^,^^32^ These were Samama school (latitude: −14.417465°; longitude: 35.217580°), Mchoka school (latitude: −14.439481°; longitude: 35.220644°), Sungusya school (latitude: −14.386472°; longitude: 35.311398°), and Malinde (St. Martin’s) school (latitude: −14.351401°; longitude: 35.294435°) (Figure 1). In total, 362 school-age children (age range: 6–16 years; mean age: 10.6 years) were surveyed across all four primary schools (Samama school, *N* = 121; Mchoka school, *N* = 121; Sungusya school, *N* = 60 and Malinde [St. Martin’s] school, *N* = 61). The number of study participants was weighted more toward schools situated on the western side of the lake based on previous parasitological surveys and anticipated prevalence’s of *G. duodenalis* infection.^3^^,^^28^^,^^29^

### Questionnaire responses.

Questionnaire responses were provided by all 362 children across all four primary schools to infer lake water consumption and cattle (cow) contact behaviors, as well as current intestinal pathology (see supplemental material). Yes–no responses were given to the following questions: Do you drink the lake [Lake Malawi] water? Do you have regular contact with livestock cattle (cows)? Do you currently have abdominal (stomach) pain? and Do you currently have loose stool (diarrhea)?

### Collection, storage, and transportation of fecal material.

One stool sample was provided by 313 (86.2%) study participants across all four primary schools (Samama school, *N* = 108; Mchoka school, *N* = 105; Sungusya school, *N* = 53; and Malinde [St. Martin’s] school, *N* = 47), (age range: 6–16 years; mean age: 10.8 years). A subset of fecal material from each stool sample was used for RDT testing as required. In addition, approximately 2 g of fecal material from each stool sample was filtered through a standard 212-µM gauge filter and stored within a labeled 2-mL screwcap tube containing 1 mL 100% ethanol for preservation and transportation. All RDT testing and fecal preservation in ethanol was carried out no more than 9 hours after stool sample provision. All ethanol-preserved fecal material was transported to the LSTM under ambient conditions for DNA extraction and genetic analyses.

### Rapid diagnostic test screening.

#### *Giardia/Cryptosporidium* QUIK CHEK RDT.

*Giardia*/*Cryptosporidium* QUIK CHEK lateral-flow RDTs were used to diagnose infection with *G. duodenalis,* according to manufacturer’s instructions. This highly portable immunoassay is used to detect *G. duodenalis*–specific cyst cell-surface antigens excreted within the feces of an infected host.^27^^,^^39^^,^^40^ QUIK CHEK diagnosis was carried out using fecal material from a total of 312 stool samples across all four schools (Samama school, *N* = 108; Mchoka school, *N* = 104; Sungusya school, *N* = 53; and Malinde [St. Martin’s] school, *N* = 47). QUIK CHEK RDTs that did not present a visible test band were deemed negative and scored as 0, whereas QUIK CHEK RDTs that presented a visible test band were deemed positive and scored according to band strength: light infection (faint band), 1; moderate infection (moderate band), 2; heavy infection (strong band), 3.

#### Fecal occult blood GP/Pro RDT.

Fecal occult blood (FOB) GP/Pro lateral-flow RDTs (Home Health, UK) were used to screen for any overt presence of blood within the feces of study participants (used as a marker of intestinal damage and pathology), according to manufacturer’s instructions. The FOB-RDT was carried out using fecal material from 199 stool samples across two schools (Samama school, *N* = 105; Mchoka school, *N* = 94). FOB-RDTs that did not present a visible test band were deemed negative and scored as 0, whereas FOB-RDTs that presented a visible test band were deemed positive and scored according to band strength: light outcome (faint band), 1; moderate outcome (moderate band), 2; heavy outcome (strong band), 3.

#### Fecal calprotectin GP/Pro RDT.

Fecal calprotectin (FC) GP/Pro lateral-flow RDTs (Home Health, Watford, UK) were used to screen for any overt presence of calprotectin within the feces of study participants (also used as a marker of intestinal damage and pathology), according to manufacturer’s instructions. The FC-RDT was carried out using fecal material from 98 stool samples provided by study participants at Samama school. FC-RDTs that did not present a visible test band were deemed negative and scored as 0, whereas FC-RDTs that presented a visible test band were deemed positive and scored according to band strength: light outcome (faint band), 1; moderate outcome (moderate band), 2; heavy outcome (strong band), 3.

### Molecular diagnosis and taxon assemblage typing.

The genetic loci used most extensively to characterize *G. duodenalis* assemblage types are found within the 18S small subunit ribosomal ribonucleic acid (SSU rRNA) and within genes coding for triosephosphate isomerase (*tpi*) enzymes, glutamate dehydrogenase (*gdh*) enzymes, and β-giardin (*bg*) proteins.^11^^,^^35^^,^^41^^,^^42^ It has been shown, however, that assemblage types can occasionally be discordant between molecular targets.^11^^,^^43^ A multilocus genotyping approach (i.e., targeting at least two loci to confirm assemblage type) is therefore recommended.^43^^–^^46^ As such, here, after DNA extraction from ethanol-preserved fecal samples and an initial diagnostic 18S real-time PCR to identify *G. duodenalis*–positive samples,^34^ we aimed to genotype all *G. duodenalis* infections to assemblage level using both nested PCR targeting the *bg* locus^35^ and a confirmatory *G. duodenalis* assemblage A- and B-specific duplex real-time PCR assay targeting the *tpi* locus.^36^

#### DNA extraction.

DNA was isolated from each ethanol-preserved fecal sample using the QIAamp DNA mini kit (Qiagen, Hilden, Germany) according to manufacturer’s instructions with minor revisions including a bead-beating step using a MagNA Lyser cell disrupter (Roche Applied Science, Penzberg, Germany) as detailed previously.^47^ During DNA extraction, phocine herpes virus 1 (PhHV-1) was added to an ATL buffer and proteinase K mix (2 μL PhHV-1 per sample) to act as an internal DNA extraction control.^48^^,^^49^ DNA extractions were performed in batches of 23 fecal samples. Each batch also included one DNA extraction-negative control sample that was subjected to the same DNA extraction protocol but did not contain any fecal material. All DNA extraction-negative samples were screened for presence of *G. duodenalis* and PhHV-1 DNA during an initial diagnostic 18S real-time PCR, as detailed subsequently.

#### Diagnostic real-time PCR detection of *G. duodenalis* 18S ribosomal RNA.

Using all 310 fecal DNA extract samples, a duplex diagnostic real-time PCR was carried out to detect and amplify a multicopy 62-bp *G. duodenalis*–specific nuclear 18S rRNA region within the small subunit ribosomal ribonucleic acid (SSU RNA),^34^ as well as a 89-bp fragment of the PhHV-1 glycoprotein B gene to ensure successful DNA extraction. Details of primer/probe sequences, reaction mix used, and PCR conditions can be found in Supplemental Table 1A–C. This real-time PCR assay included one *G. duodenalis* positive control using 5 μL of *G. duodenalis* template DNA (extracted from a fecal sample provided by a heavily infected individual excreting ∼1,*000 G. duodenalis* cysts per gram of feces; as estimated using stool microscopy^5^^,^^27^), one DNA extraction-negative control using 5 μL of template DNA taken from a DNA extraction sample that did not contain any fecal material, and one no-template-negative control using 5 μL ddH_2_O in place of template DNA.

All real-time PCR assays were carried out using a Magnetic Induction Cycler (MIC) PCR thermocycler (Bio Molecular Systems, Upper Coomera, Australia). All real-time PCR assay plates were prepared using a Myra liquid handling system (Bio Molecular Systems, London, UK). A cycle threshold (Ct) value of ≤ 39 was deemed positive for infection with *G. duodenalis*, whereas a Ct value of ≥ 40 was deemed negative for infection with *G. duodenalis*.^34^ The intensity of *G. duodenalis* infection was categorized according to the following Ct values: light infection, 30–39; moderate infection, 20–29; and heavy infection, ≤ 19.^34^ For quality assurance, 10% of *G. duodenalis*–positive and 10% of *G. duodenalis*–negative samples were retested. Any samples that did not amplify PhHV-1 DNA were tested again within a secondary repeat screen. Any samples that did not amplify PhHV-1 DNA during either PCR were considered to have failed DNA extraction and so were not included in any further analysis.

#### Nested PCR, genotyping and phylogenetic analysis of the *G. duodenalis bg* locus.

Using all fecal DNA extract isolates deemed positive for *G. duodenalis* infection during the initial diagnostic 18S real-time PCR screen described earlier, a nested PCR was carried out to detect and amplify a single-copy 511-bp region of the *G. duodenalis bg* locus. This was then used for genotyping and phylogenetic analysis according to a previously published methodology,^35^ with minor modifications. Nested PCR was used to limit nonspecific binding of PCR products and so reduce potential amplification of nontarget regions and to compensate for the *bg* locus being only single copy. Details of all primer sequences, both reaction mixes used, and both initial and nested PCR conditions can be found in Supplemental Table 2A–C, respectively. The initial PCR included a positive control using 2 μL of reference *G. duodenalis* template DNA (extracted from a fecal sample provided by a heavily infected individual excreting ∼1,*000 G. duodenalis* cysts per gram of feces; as estimated using stool microscopy,^5^^,^^27^ as well as a nontemplate negative control using 2 μL ddH_2_O in place of template DNA. Successful amplification was verified by running 5 μL of nested PCR product on a 1.5% agarose gel stained with SYBR safe gel stain (ThermoFisher Scientific, Waltham, MA).

Nested PCR products were purified using the ExoSAP-IT PCR Product Clean-Up Reagent (ThermoFisher Scientific) according to manufacturer’s instructions and were then sequenced in both forward and reverse directions using Sanger sequencing.^5^ Obtained chromatograms were visualized and nucleotide sequences were trimmed/masked and edited using Geneious Prime version 2023.01 (Biomatters, Auckland, New Zealand). Any poor-quality sequences, defined as chromatograms with excessive background noise, were omitted from any analysis.

*G. duodenalis* assemblages were identified by carrying out nucleotide Basic Local Alignment Search Tool (BLAST) searches within the NCBI database^50^ using each cleaned sequence in turn against both *G. duodenalis* assemblages A and B in tandem. To check whether any sequences were more closely related to *G. duodenalis* assemblages C through H, additional nucleotide BLAST searches were carried out using each cleaned sequence in turn against the *G. duodenalis* (non–assemblage-specific) database. Nucleotide BLAST hits with the greatest match and query cover scores as well as lowest E-values were considered the most closely related assemblage and the sequence of each most closely related BLAST hit was downloaded from GenBank in FASTA format to serve as an assemblage-specific reference sequence. Five randomly selected *G. duodenalis* assemblage A forward sequences and five randomly selected *G. duodenalis* assemblage B forward sequences were uploaded to the GenBank repository^51^ (Accession numbers: OR228449 - OR228453 and OR228454 - OR228458, respectively).

To identify any samples co-infected with both *G. duodenalis* assemblages A and B, a multiple alignment with fast Fourier transform (MAFFT) alignment was carried out using all obtained assemblage A and B reference sequences (*N* = 8 and 8, respectively) within Geneious Prime (default MAFFT alignment parameter settings). This MAFFT alignment was then visualized within Genious Prime to identify which nucleotide positions showed single nucleotide polymorphism (SNP) variations between both assemblages. Chromatograms of all study sample sequences (forward direction and grouped according to school site) were then MAFFT aligned to this existing reference alignment using Geneious Prime and visualized at SNP positions to check for double-peaking with both assemblage-specific nucleotides, suggesting co-infection with both A and B assemblages.

To infer evolutionary relationships between all study sequences identified as single-assemblage infections, all eight reference sequences and all sequences identified as mixed-assemblage infections were removed from this MAFFT alignment, which was then exported from Geneious Prime and imported into PopART version 1.7.^52^ A Templeton Crandall and Sing (TCS) haplotype network^53^ was then constructed and annotated within PopART.^52^

#### Real-time PCR detection of *G. duodenalis* assemblage A- and B-specific DNA loci.

Again, using all fecal DNA extract isolates deemed positive for *G. duodenalis* infection during the initial diagnostic 18S real-time PCR screen described earlier, a duplex real-time PCR was carried out to detect and amplify *G. duodenalis* assemblage A- and B-specific fragments within the *tpi* locus (both single-copy targets 77-bp in length) according to previous work^35^^,^^36^ with minor modifications. This was used to detect single-assemblage (A or B) and mixed-assemblage (A and B) infections and served as a confirmatory screen to check for any assemblage dichotomy between *bg* and *tpi* loci. Details of primer/probe sequences, reaction mix used, and PCR conditions can be found in Supplemental Table 3A–C. This real-time PCR assay included three positive controls: one using 3 μL of reference *G. duodenalis* assemblage A template DNA, one using 3 μL of reference *G. duodenalis* assemblage B template DNA, and one using 3 μL of reference *G. duodenalis* mixed-assemblage A and B template DNA (as determined previously^5^), as well as one negative control using 3 μL ddH_2_O in place of template DNA.

All real-time PCR assays were carried out using a MIC PCR thermocycler (Bio Molecular Systems, London, UK). All real-time PCR assay plates were prepared using a Myra liquid handling system (Bio Molecular Systems). A Ct value ≤ 45 was deemed positive for infection with either *G. duodenalis* assemblage A or B, whereas a Ct value of ≥ 46 was deemed either negative for infection with either *G. duodenalis* assemblage or failed PCR detection and amplification.^5^^,^^36^ For quality assurance, 10% of *G. duodenalis* assemblage A positive, 10% of *G. duodenalis* assemblage B positive, 10% of *G. duodenalis* assemblage A and B positive, and 10% of samples that failed to amplify the *G. duodenalis* assemblage-specific *tpi* fragments were retested.

### Statistical analysis.

All statistical analyses and generation of figures were caried out within R Studio version 2021.09.0, build 351 (Posit, San Francisco, CA),^38^ unless stated otherwise.

#### *Giardia duodenalis* diagnostic 18S real-time PCR Ct values and participant age.

Any association between diagnostic 18S real-time PCR Ct values and participant age was measured using a Spearman’s rank coefficient test. This was calculated using the cor.test function as part of the R stats package.^38^

#### Diagnostic outcome of RDTs compared with that of diagnostic 18S real-time PCR.

Diagnostic outcomes (positive/negative) of QUIK CHEK, FOB- and FC-RDTs were compared with that of the diagnostic 18S real-time PCR. Sensitivity, specificity, positive predictive, and negative predictive values were calculated using the epi.tests function as part of the epiR package version 2.0.39.^54^ In addition, any association between QUIK CHEK RDT band strength (light infection, moderate infection, and heavy infection) and diagnostic 18S real-time PCR Ct values was also measured using a Spearman’s rank coefficient test. This was calculated using the cor.test function as part of the R stats package.^38^

#### Measuring any associations between *G. duodenalis* infection status and intestinal pathology.

Odds ratios (OR) were calculated to measure any association between self-reported consumption of lake water (as ascertained by yes–no questionnaire responses) and infection with *G. duodenalis* (as ascertained by diagnostic 18S real-time PCR outcome). Odds ratios were also calculated to measure any association between self-reported regular contact with livestock cattle (cows; as ascertained by yes–no questionnaire responses), and infection with *G. duodenalis* (as ascertained by diagnostic 18S real-time PCR outcome). In addition, ORs were calculated to measure any association between infection with *G. duodenalis* (as ascertained by diagnostic 18S real-time PCR outcome) and presence of FOB (according to FOB-RDT outcomes), self-reporting of abdominal (stomach) pain, and self-reporting of loose stool (diarrhea).

Odds ratios were also calculated to measure any association between infection with *G. duodenalis* assemblage A (either single assemblage A infection or mixed assemblage A and B infection versus no infection and single assemblage B infection) and self-reported consumption of lake water, as well as self-reported regular contact with livestock cattle. Likewise, ORs were calculated in this manner to measure any association between infection with *G. duodenalis* assemblage B and self-reported consumption of lake water, as well as self-reported regular contact with livestock cattle.

Furthermore, ORs were calculated to measure any association between single infection with *G. duodenalis* assemblage A (versus no infection), and presence of FOB (according to FOB-RDT outcomes), self-reporting of abdominal (stomach) pain, and self-reporting of loose stool (diarrhea). Likewise, ORs were calculated in this manner to measure any association between single infection with *G. duodenalis* assemblage B and presence of FOB (according to FOB-RDT outcomes), self-reporting of abdominal (stomach) pain, and self-reporting of loose stool (diarrhea). These analyses were restricted only to single-assemblage infections versus no infection only (and not to the mixed-assemblage infections) because mixed-assemblage infections may confound pathological outcomes. Finally, ORs were also calculated to measure any association between mixed infection with both *G. duodenalis* assemblages A and B and presence of FOB (according to FOB-RDT outcomes), self-reporting of abdominal (stomach) pain, and self-reporting of loose stool (diarrhea). Odds ratios were calculated using the oddsratio function as part of the epitools package version 0.5.10.1.^55^

## RESULTS

### Questionnaire responses.

The total number of questionnaire respondents also assessed for *G. duodenalis* infection using the diagnostic 18S real-time PCR across all four schools was 305. Of these participants, responses to all questions are provided in Table 1.

**Table 1:** Questionnaire responses given by participants assessed for *Giardia duodenalis* infection using the diagnostic 18S real-time polymerase chain reaction across all four schools (*N* = 305)

Question	Responded yes	Responded no
Do you drink the lake [Lake Malawi] water?	187 (63%)	122 (37%)
Do you have regular contact with livestock cattle (cows)?	166 (54%)	139 (46%)
Do you currently have abdominal (stomach) pain?	135 (44%)	170 (56%)
Do you currently have loose stool (diarrhea)?	98 (32%)	207 (68%)

The total number of questionnaire respondents that were either deemed negative for infection using the diagnostic 18S real-time PCR or that had positive infection status (single infection with *G. duodenalis* assemblage A, single infection with *G. duodenalis* assemblage B, or mixed infection with both *G. duodenalis* assemblages A and B) identified was 289. Of these participants, responses to the questions “do you currently have abdominal (stomach) pain?” and “do you currently have loose stool (diarrhea)?” can are provided in Table 2.

**Table 2:** Questionnaire responses given by participants that were either deemed negative for infection using the diagnostic 18S real-time polymerase chain reaction or that had positive infection status (single infection with *Giardia duodenalis* assemblage A, single infection with *G. duodenalis* assemblage B, or mixed infection with both *G. duodenalis* assemblages A and B) identified (*N* = 289)

Question	Responded yes	Responded no
Do you currently have abdominal (stomach) pain?	128 (44%)	161 (56%)
Do you currently have loose stool (diarrhea)?	92 (32%)	197 (68%)

### Rapid diagnostic test screening.

#### *Giardia/Cryptosporidium* QUIK CHEK RDT.

A total of 109 (34.9% of 312) participants were deemed positive for *G. duodenalis* infection using the QUIK CHEK RDT (Samama school 36% prevalence; Mchoka school 35% prevalence; Sungusya school 34% prevalence; and Malinde [St. Martin’s] school 34% prevalence). Of these, 16 (5% of all participants; 15% of positive infections) were deemed light infections; 50 (15% of all participants; 45% of positive infections) were deemed moderate infections; and 42 (13% of all participants; 39% of positive infections) were deemed heavy infections.

#### Fecal occult blood GP/Pro RDT.

A total of 56 (27% of 207) participants were deemed positive for overt FOB using the FOB-RDT. Of these, one (0.5% of all participants; 1.8% of positive outcomes) was deemed light outcome; 47 (23% of all participants; 84% of positive outcomes) were deemed moderate outcome; and eight (7% of all participants; 14.2% of positive outcomes) were deemed heavy outcome.

#### Fecal calprotectin GP/Pro RDT.

A total of 12 (12% of 101 participants) were deemed positive for overt FC using the FC-RDT. Of these, none were deemed light outcome; eight (8% of all participants; 67% of positive outcomes) were deemed moderate outcome; and four (4% of all participants; 33% of positive outcomes) were deemed heavy outcome.

### Molecular diagnosis and *G. duodenalis* assemblage typing.

DNA extraction was performed using 310 of all 313 (99%) ethanol-preserved fecal samples because three fecal samples were deemed too low in volume to carry out DNA extraction.

#### Diagnostic real-time PCR detection of *G. duodenalis* 18S ribosomal RNA.

Of the 310 samples screened, five failed to amplify the PhHV-1 internal DNA extraction control in both initial and repeat screens and were therefore omitted from further analysis. Of the remaining 305 samples, 120 (39.3%) were deemed positive for *G. duodenalis* infection using the diagnostic 18S real-time PCR (Samama school, 45% prevalence; Mchoka school, 37% prevalence; Sungusya school, 34% prevalence; and Malinde [St. Martin’s], school 35% prevalence). Of all fecal DNA isolates deemed positive for *G. duodenalis* infection, 54 (18% of all participants; 45% of positive infections) were deemed light infections; 54 (18% of all participants; 45% of positive infections) were deemed moderate infections; and 12 (4% of all participants; 10% of positive infections) were deemed heavy infections, as ascertained by diagnostic 18S real-time PCR Ct values. The diagnostic outcome of all assay control samples was as expected. All samples retested for quality assurance purposes (10% *G. duodenalis* positive and 10% *G. duodenalis* negative) gave the same diagnostic outcome as the initial screen with *G. duodenalis–*positive samples giving a Ct value within ± Ct 5 given during the initial screen.

#### *Nested PCR, genotyping, and phylogenetic analysis of the* G. duodenalis bg *locus.*

The 511-bp region of the *G. duodenalis bg* locus was successfully amplified in 108 samples (Samama school, *N* = 42; Mchoka school, *N* = 35; Sungusya school, *N* = 17 and Malinde [St. Martin’s] school, *N* = 14), all of which were then purified and sequenced in both forward and reverse directions using Sanger sequencing. One sample, provided by a study participant attending Mchoka school, was omitted from any further analysis because this was deemed a poor-quality sequence. As such, a total of 107 samples (89% of all samples deemed positive using diagnostic 18S real-time PCR; Samama, school *N* = 42; Mchoka school, *N* = 34; Sungusya school, *N* = 17; and Malinde (St. Martin’s), school *N* = 14) were used for genotyping and phylogenetic analysis. The diagnostic outcome of all assay control samples was as expected.

**Figure 2.**
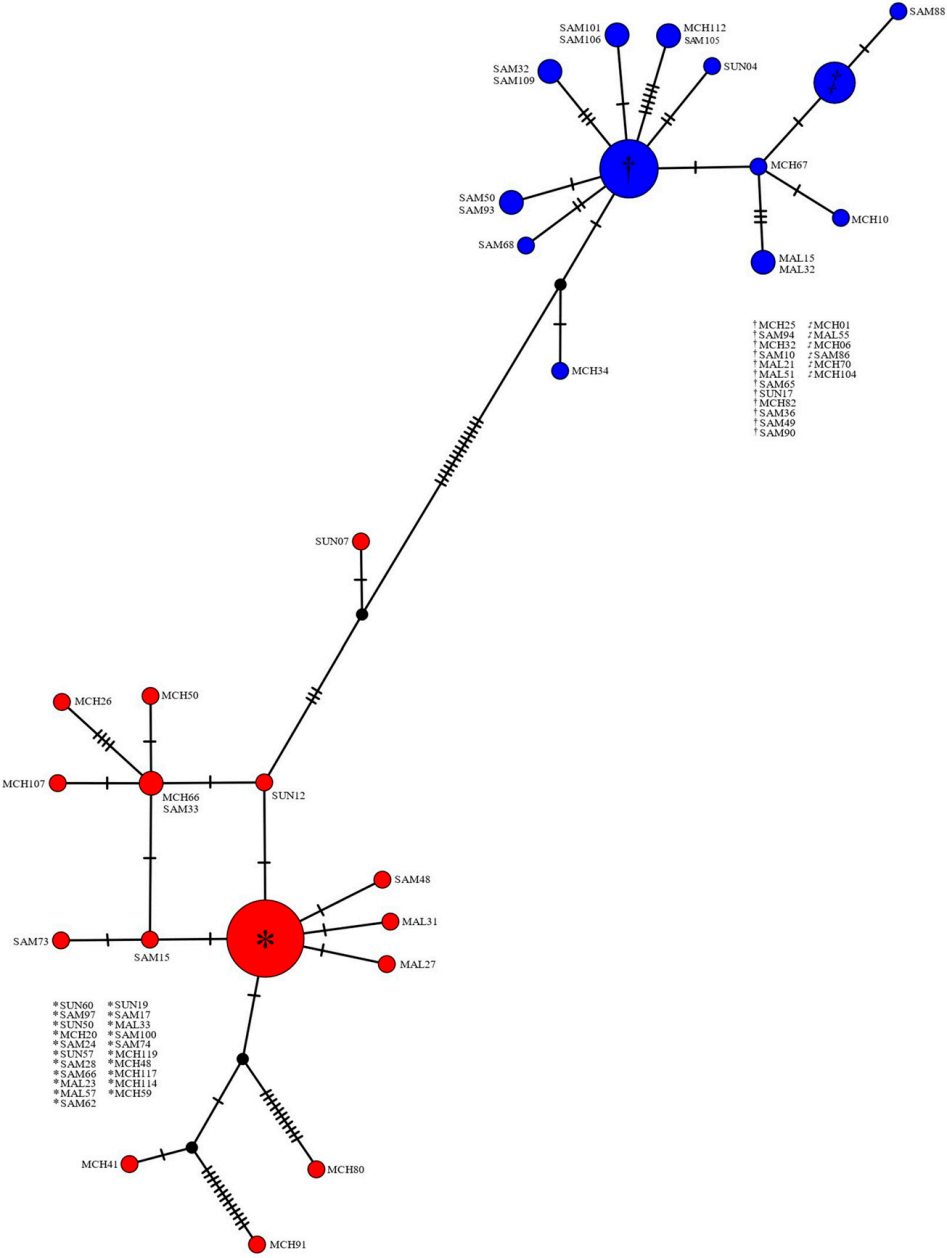
Templeton Crandall and Sing haplotype network of *Giardia duodenalis* β-giardin (*bg*) locus (forward direction sequences). Each node (circle) represents a unique haplotype. Nodes colored red denote *G. duodenalis* assemblage A haplotypes. Nodes colored blue donate *G. duodenalis* assemblage B haplotypes. Nodes colored black denote a missing but predicted ancestral haplotype. Size of nodes is proportional to the frequency of each haplotype. Hatched lines denote the number of single nucleotide polymorphisms between nodes. MAL = Malinde (St. Martin’s) school; MCH = Mchoka school; SAM = Samama school; SUN = Sungusya school.

When targeting the *G. duodenalis bg* locus, 38 samples (35% of all samples with infectious status identified using *bg* locus; 13% of all samples screened using diagnostic 18S real-time PCR) were identified as single *G. duodenalis* assemblage A infections (Samama school, *N* = 10; Mchoka school, *N* = 16; Sungusya school, *N* = 7; and Malinde [St. Martin’s] school, *N* = 5), whereas 34 samples (32% of all samples with infectious status identified using *bg* locus; 11% of all samples screened using diagnostic 18S real-time PCR) were identified as single *G. duodenalis* assemblage B infections (Samama school, *N* = 18; Mchoka school, *N* = 11; Sungusya school, *N* = 1; and Malinde [St. Martin’s] school, *N* = 4). A total of 35 samples (33% of all samples with infectious status identified using *bg* locus; 11% of all samples screened using diagnostic 18S real-time PCR) were identified as mixed *G. duodenalis* assemblage A and B infections (Samama school, *N* = 14; Mchoka school, *N* = 7; Sungusya school, *N* = 9; and Malinde [St. Martin’s], school *N* = 5). No samples were identified as *G. duodenalis* assemblages C-H. A TCS haplotype network constructed using all MAFFT aligned study sequences (*bg* locus; forward direction) identified as single-assemblage infections can be seen in Figure 2.

Twenty-eight unique *G. duodenalis bg* loci haplotypes were identified within this study group, 15 of which were associated with *G. duodenalis* assemblage A and 13 with *G. duodenalis* assemblage B. There is clear and distinct divergence between *G. duodenalis* assemblages A and B, haplotypes of which are separated by 14 SNPs. Twenty-one of 38 (55%) sequences identified as *G. duodenalis* assemblage A shared a common haplotype and two of 38 (5%) also shared a common haplotype. The remaining *15 G. duodenalis* assemblage A sequence haplotypes were unique and not shared. However, one sequence (SUN07) was more closely related to *G. duodenalis* assemblage B than the remaining *37 G. duodenalis* assemblage A sequences, and two sequences (MCH80 and MCH91) were far more genetically distinct than the remaining *36 G. duodenalis* assemblage A sequences (separated from the most common *G. duodenalis* assemblage A haplotype by 12 and 15 SNPs, respectively). Likewise, 11 of 34 (32%) sequences identified as *G. duodenalis* assemblage B shared one common haplotype, and six of 34 (18%) also shared a common haplotype. Five sequences each shared a common haplotype with one other sequence, and six *G. duodenalis* assemblage B sequence haplotypes were unique and not shared. One sequence (MCH34) was more closely related to *G. duodenalis* assemblage A than the remaining *33 G. duodenalis* assemblage B sequences, albeit by only two SNPs. All four schools were well represented within haplotypes across both *G. duodenalis* assemblages, and so no evidence of *G. duodenalis* divergence by locality (school site) was suggested.

#### *Real-time PCR detection of* G. duodenalis *assemblage A- and B-specific DNA loci.*

The 77-bp region of the *G. duodenalis tpi* locus was successfully amplified in 78 samples (Samama school, *N* = 33; Mchoka school, *N* = 21; Sungusya school, *N* = 14; and Malinde [St. Martin’s] school, *N* = 10), representing 65% of all samples deemed positive for *G. duodenalis* infection by diagnostic 18S real-time PCR and 55% of samples with infection status identified using nested PCR/Sanger sequencing (targeting the *bg* locus). When targeting the *G. duodenalis tpi* locus, 20 samples (26% of all samples with infectious status identified using *tpi* locus; 17% of positive infections using diagnostic 18S real-time PCR) were identified as single *G. duodenalis* assemblage A infections (Samama school, *N* = 8; Mchoka school, *N* = 7; Sungusya school, *N* = 3; and Malinde [St. Martin’s] school *N* = 2), whereas 45 samples (58% of all samples with infectious status identified using *tpi* locus; 38% of positive infections using diagnostic 18S real-time PCR) were identified as single *G. duodenalis* assemblage B infections (Samama school, *N* = 20; Mchoka school, *N* = 11; Sungusya school, *N* = 7; and Malinde [St. Martin’s], school *N* = 7). A total of 13 (17% of all samples with infectious status identified using *tpi* locus; 11% of positive infections using diagnostic 18S real-time PCR) samples were identified as mixed *G. duodenalis* assemblage A and B infections (Samama school, *N* = 5; Mchoka school, *N* = 3; Sungusya school, *N* = 4 and Malinde [St. Martin’s], school *N* = 1). The diagnostic outcome of all assay control samples was as expected. All samples retested for quality assurance purposes (10% of *G. duodenalis* assemblage A positive, 10% of *G. duodenalis* assemblage B positive, 10% of *G. duodenalis* assemblage A and B positive, and 10% of samples that failed to amplify the *G. duodenalis* assemblage-specific *tpi* fragments) gave the same diagnostic outcome as the initial screen with *G. duodenalis–*positive samples giving a Ct value within ± Ct 5 given during the initial screen.

#### Comparing *G. duodenalis* assemblage types as identified when targeting bg and tpi loci.

In total, *18 G. duodenalis* infections identified as single assemblage A when targeting the *bg* locus were also identified as single assemblage A when targeting the *tpi* locus. One infection identified as single assemblage A when targeting the *bg* locus was identified as mixed-assemblage A and B when targeting the *tpi* locus, and two infections identified as mixed-assemblage A and B when targeting the *bg* locus were identified as single assemblage A when targeting the *tpi* locus. The remaining 19 infections identified as single assemblage A when targeting the *bg* locus could not be identified when targeting the *tpi* locus due to failure in amplifying the *G. duodenalis* assemblage A-specific *tpi* fragment using the duplex *G. duodenalis* assemblage A- and B-specific real-time PCR.

Likewise, 28 of *G. duodenalis* infections identified as single assemblage B when targeting the *bg* locus were also identified as single assemblage B when targeting the *tpi* locus. No infections identified as single assemblage B when targeting the *bg* locus identified as mixed-assemblage A and B when targeting the *tpi* locus, and 16 infections identified as mixed-assemblage A and B when targeting the *bg* locus were identified as single assemblage B when targeting the *tpi* locus. The remaining six infections identified as single assemblage B when targeting the *bg* locus could not be identified when targeting the *tpi* locus due to failure in amplifying the *G. duodenalis* assemblage B-specific *tpi* fragment using the duplex *G. duodenalis* assemblage A- and B-specific real-time PCR. No discordance between assemblage-type was found between *bg* and *tpi* loci across all single-assemblage infections successfully identified using both targets.

In addition, 12 of *G. duodenalis* infections identified as mixed assemblage A and B infections when targeting the *bg* locus were also identified as mixed assemblage A and B infections when targeting the *tpi* locus. The remaining 23 of these infections could not be identified as mixed-assemblage infections due to failure in amplifying either or both *G. duodenalis* assemblage A- or assemblage B-specific *tpi* fragments using the duplex *G. duodenalis* assemblage A- and B-specific real-time PCR. Of these 23 infections that could not be identified as mixed infections using the *G. duodenalis* assemblage A- and B-specific real-time PCR, two were identified as single-infection assemblage A (as assemblage B DNA was not detected), and 16 were identified as single-infection assemblage B (as assemblage A DNA was not detected). Furthermore, neither *G. duodenalis* assemblage A or B DNA was detected in five samples deemed mixed-assemblage infections (by nested PCR/Sanger sequencing targeting the *bg* locus) when using the *G. duodenalis* assemblage A- and B-specific real-time PCR (targeting *tpi* loci).

**Figure 3.**
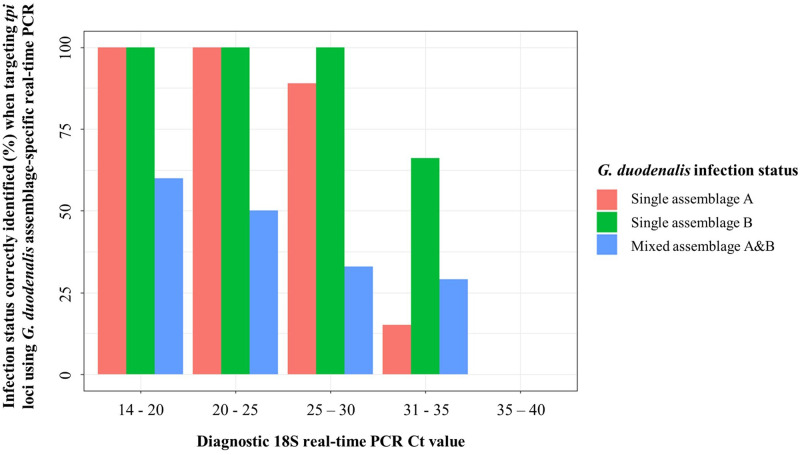
*Giardia duodenalis* infection status correctly identified (%) when targeting triosephosphate isomerase (*tpi*) loci using *G. duodenalis* assemblage-specific real-time polymerase chain reaction PCR compared with when targeting β-giardin (*bg*) locus using nested PCR with subsequent Sanger sequencing, relevant to diagnostic 18S real-time PCR Ct values. Plot generated using ggplot2 package version 3.4.0.^56^

When evaluating the performance of the *G. duodenalis* assemblage-specific real-time PCR assay (targeting *tpi* loci) compared with nested PCR/Sanger sequencing (targeting the *bg* locus) and relative to diagnostic 18S real-time PCR Ct values, sensitivity of the assay appears to decrease as diagnostic 18S real-time PCR Ct values increase. This suggests that *G. duodenalis* assemblages in samples with a lower concentration of DNA (potentially caused by a lower intensity of infection), may be less likely to be correctly identified when targeting the *tpi* region using this real-time PCR assay (Figure 3).

**Figure 4.**
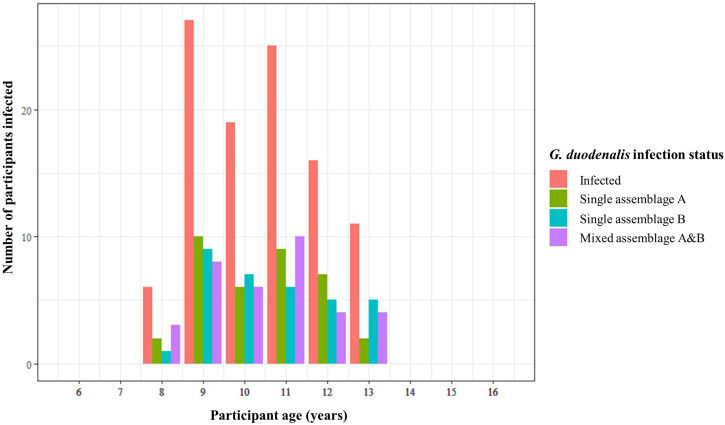
Bar chart showing number of participants infected with *Giardia duodenalis* according to diagnostic 18S real-time polymerase chain reaction across all participant ages, as well as number of participants harboring single infections with *G. duodenalis* assemblage A, single infections with *G. duodenalis* assemblage B, and mixed infections with both *G. duodenalis* assemblages A and B, across all participant ages when targeting the β-giardin locus. Plot generated using ggplot2 package version 3.4.0.^56^

The *G. duodenalis* assemblage-specific real-time PCR assay (targeting *tpi* loci), appears reliable when screening single-assemblage (A or B) infections between diagnostic 18S real-time PCR Ct values 14 to ∼30. However, this assay appears unreliable when screening single-assemblage infections when diagnostic 18S real-time PCR Ct values are > 30, particularly when screening samples identified as single *G. duodenalis* assemblage A infections by nested PCR/Sanger sequencing (targeting the *bg* locus). In addition, although performance is slightly improved with lower diagnostic 18S real-time PCR Ct values, when screening mixed-assemblage (A and B) infections, the assay appears unreliable regardless of diagnostic 18S real-time PCR Ct value. This is most likely because of the assay’s poor performance in detecting and amplifying *G. duodenalis* assemblage A DNA (Figure 4).

#### *Giardia duodenalis* assemblage infection status and participant age.

When using the diagnostic 18S real-time PCR, *G. duodenalis* infection was detected within study participants aged between 8 and 13 years. No participants aged 6 and 7 or 14 to 16 were identified as infected. Participants aged between 9 and 11 years comprised 68.3% of total infections, whereas participants aged 8, 12, and 13 years comprised 31.7% of total infections (Figure 4). Single-assemblage infections (regardless of assemblage type) and mixed-assemblage infections followed a similar pattern, and so there did not appear to be any associations between participant age and assemblage infection status (Figure 4).

### Statistical analysis.

#### G. duodenalis *diagnostic 18S real-time PCR Ct values and participant age.*

No association between participant age and diagnostic 18S real-time PCR Ct values was found (*R* = −0.006, *P* = 0.47).

#### Diagnostic outcome of RDTs compared with that of diagnostic 18S real-time PCR.

**Figure 5.**
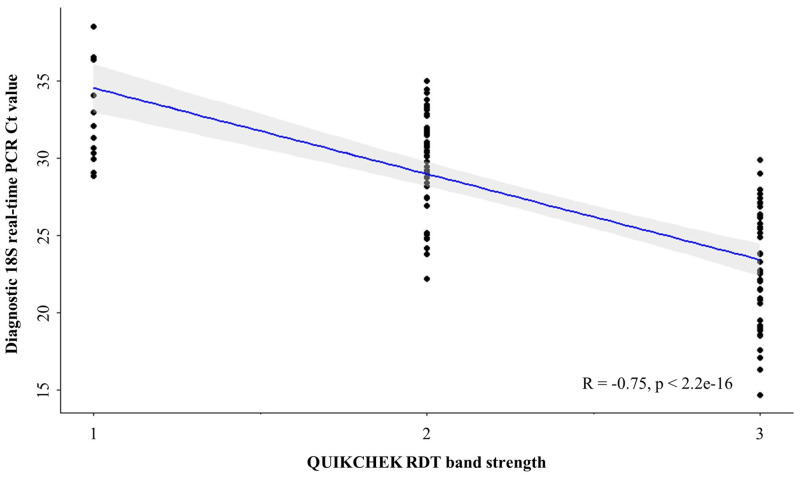
Scatterplot showing *Giardia duodenalis* diagnostic 18S real-time polymerase chain reaction values against QUIK CHEK RDT band strength. Regression line (Spearman’s rank coefficient) is plotted in blue, and 95% ICs can be seen shaded gray. Plot generated using ggplot2 package version 3.4.0.^56^

When comparing diagnostic outcomes of the QUIK CHEK RDT against the diagnostic 18S real-time PCR, QUIK CHEK had a sensitivity of 83% (95% CI: 75–90), a specificity of 96% (95% CI: 92–98), a positive predictive value of 93% (95% CI: 87–97), and a negative predictive value of 90% (95% CI: 85–94). In addition, there was a strong negative and significant correlation between QUIK CHEK RDT band strength and diagnostic 18S real-time PCR Ct values (r = −0.75, *P* = 2.2 e-16) (Figure 5). There was no notable difference in diagnostic performance of the QUIK CHEK RDT between *G. duodenalis* assemblages A and B, when compared with diagnostic 18S real-time PCR.

When comparing diagnostic outcomes of the FOB-RDT against the diagnostic 18S real-time PCR, FOB-RDT had a sensitivity of 28% (95% CI: 18–39), a specificity of 77% (95% CI: 68–84), a positive predictive value of 46% (95% CI: 32–61), and a negative predictive value of 60% (95% CI: 51–68). When comparing diagnostic outcomes of the FC-RDT against the diagnostic 18S real-time PCR, FC-RDT had a sensitivity of 6% (95% CI: 1–17), a specificity of 82% (95% CI: 69–91), a positive predictive value of 25% (95% CI: 5–57), and a negative predictive value of 48% (95% CI: 37–59).

#### *Measuring any associations between* G. duodenalis *infection status and intestinal pathology.*

There was a statistically insignificant and weak positive association between self-reported consumption of lake water and infection with *G. duodenalis* (OR: 1.18, 95% CI: 0.73–1.9, *P =* 0.58). In addition, there was a statistically insignificant and weak negative association between self-reported regular contact with livestock cattle, and infection with *G. duodenalis* (OR: 0.94, 95% CI: 0.44–1.95, *P =* 0.87). There was a statistically insignificant and weak positive association between infection with *G. duodenalis* and presence of FOB (OR: 1.08, 95% CI: 0.57–2. 04, *P =* 0.8). However, there was a statistically significant and strong positive association between infection with *G. duodenalis* and both self-reporting of abdominal pain (OR: 2.1, 95% CI: 1.32–3.36, *P* = 0.002), and self-reporting of loose stool (diarrhea) (OR: 1.95, 95% CI: 1.19–3.19, *P* = 0.007).

There was a statistically insignificant and weak positive association between self-reported consumption of lake water and infection with *G. duodenalis* assemblage A (OR: 1.26, 95% CI: 0.73–2.22, *P =* 0.41). There was also a statistically insignificant and weak positive association between self-reported consumption of lake water and infection with *G. duodenalis* assemblage B (OR: 1.43, 95% CI: 0.82–2. 59, *P =* 0.21). In addition, there was a statistically insignificant and weak positive association between self-reported regular contact with livestock cattle and infection with *G. duodenalis* assemblage A (OR: 1.09, 95% CI: 0.46–2.36, *P =* 0.84). There was also a statistically insignificant and weak negative association between self-reported regular contact with livestock cattle and infection with *G. duodenalis* assemblage B (OR: 0.85, 95% CI: 0.32–1.96, *P =* 0.72).

There was a statistically insignificant and weak negative association between single infection with *G. duodenalis* assemblage A and presence of FOB (OR: 0.92, 95% CI: 0.31–2.43, *P =* 0.87) and self-reporting of abdominal pain (OR: 0.98, 95% CI: 0.47–2.08, *P =* 0.97). Additionally, there was no association between single infection with *G. duodenalis* assemblage A and self-reporting of loose stool/diarrhea (OR: 1, 95% CI: 0.43–2.16, *P =* 0.99). There was a statistically insignificant positive association between single infection with *G. duodenalis* assemblage B and presence of FOB (OR: 1.6, 95% CI: 0.64–3.81, *P =* 0.31); however, there was a statistically significant and strong positive association between single infection with *G. duodenalis* assemblage B and both self-reporting of abdominal pain (OR: 3.05, 95% CI: 1.44–6.78, *P =* 0.004), and self-reporting of loose stool/diarrhea (OR: 3.1, 95% CI: 1.46–6.66, *P =* 0.003).

There was a statistically insignificant negative association between mixed infection with both *G. duodenalis* assemblages A and B and presence of FOB (OR: 0.51, 95% CI: 0.11–1.73, *P =* 0.31) and statistically insignificant positive association between mixed infection with both *G. duodenalis* assemblages A and B and self-reporting of loose stool/diarrhea, (OR: 2.08, 95% CI: 0.97–4.39, *P =* 0.06). There was, however, a statistically significant and strong positive association between mixed infection with both *G. duodenalis* assemblages A and B and self-reporting of abdominal (stomach) pain (OR: 3.18, 95% CI: 1.51–7.05, *P =* 0.002). On the basis of assay sensitivity and specificity compared with diagnostic 18S real-time PCR, the overt presence of FC as measured by RDT was deemed a poor marker for *G. duodenalis* infection and so was omitted from OR analyses.

## DISCUSSION

Differentiation between human infections with *G. duodenalis* assemblages A and B as well as between single- and mixed-assemblage infections is essential to better understand the pathological impact of infection with either, or both, assemblages and thus also for improved disease surveillance and control.^26^ Here, we evaluated the prevalence of human giardiasis infection in 305 school-aged children attending four primary schools situated in close-proximity to Lake Malawi’s southern shoreline. In addition, we also aimed to identify the prevalence of differing *G. duodenalis* infection statuses (single *G. duodenalis* assemblage A infection, single *G. duodenalis* assemblage B infection, or mixed *G. duodenalis* assemblages A and B infection) and test for any association(s) between infection status and intestinal pathology.

In total, *G. duodenalis* infection status was identified in 107 study participants when targeting the *bg* locus. Although mixed-assemblage (A and B) *G. duodenalis* infections often appear to comprise approximately one-third of all infections^20^^,^^31^^,^^57^^–^^59^ as observed here (33% of all identified infections), previous studies have found that the prevalence of single-assemblage (A or B) infections are typically more weighted toward either *G. duodenalis* assemblage A or assemblage B.^5^^,^^15^^–^^25^ Here, however, the prevalence of single infections with each assemblage was similar (35% and 32%, respectively). There was clear and distinct genetic divergence found both between and within *G. duodenalis* assemblages A and B haplotypes (Figure 2). This divergence observed within both assemblages may be a result of genetically distinct subassemblages known to exist within both *G. duodenalis* assemblages A and B (named AI–AIII, BIII, and BIV^9^^,^^26^^,^^60^^,^^61^). However, additional and more thorough molecular analyses are needed to accurately confirm any potential subassemblages because this cannot be done accurately using the *bg* and *tpi* loci alone. In addition, all four schools were well represented within haplotypes across both *G. duodenalis* assemblages, suggesting limited focal transmission “hot spots’ are present within this study area.

When targeting *G. duodenalis* assemblage-specific *tpi* loci using a duplex real-time PCR, 55% of infections were successfully typed compared with when targeting the *bg* locus using nested PCR with subsequent Sanger sequencing. The *tpi*-targeting real-time PCR assay had poor sensitivity when diagnostic 18S real-time PCR Ct values were > 30 (particularly when targeting the *G. duodenalis* assemblage A *tpi* locus) and when attempting to confirm mixed-assemblage infections, regardless of diagnostic 18S Ct value (again, predominantly as a result of poor success in detecting and amplifying the *G. duodenalis* assemblage A *tpi* locus). Although real-time PCR offers a more rapid and less expensive means of assemblage typing *G. duodenalis* infections (through real-time interpretation of results and omitting the need to sequence PCR products), further work is needed to optimize and improve this real-time PCR assay, especially when attempting to genotype low-intensity single-assemblage infections or mixed infections where the intensity of infection of each individual assemblage may also be low.^62^ Until improved, the use of PCR approaches that compensate for the *tpi* locus being only single-copy, such as nested PCR (as used when targeting the *bg* locus here and when targeting the *tpi* locus elsewhere^14^^,^^62^^,^^63^) or through amplicon cloning (also used when targeting the *tpi* locus elsewhere^62^), are recommended when attempting to type *G. duodenalis* assemblage-specific *tpi* loci. When compared with diagnostic 18S real-time PCR, the *Giardia* QUIK CHEK RDT proved extremely reliable, both in end-point sensitivity and specificity, as well as when measuring the intensity of *G. duodenalis* infections (as ascertained by RDT band strength). Whilst further and more thorough assessment of the assay is needed to confirm diagnostic performance (e.g., through comparing QUIK CHEK to fecal microscopy in tandem with diagnostic 18S real-time PCR), the assay showed excellent promise as a rapid and reliable diagnostic tool capable of accurately diagnosing *G. duodenalis* infections in resource-limited settings. Both FOB and FC RDTs were deemed unreliable as a means of measuring intestinal pathology caused by *G. duodenalis* infection because no association between the presence of FOB or FC and *G. duodenalis* infection was found when using these assays.

Surprisingly, no association was found between self-reported consumption of lake water, as well as self-reported regular contact with livestock cattle, and *G. duodenalis* infection. There was also no association found between self-reporting of these behaviors and infection with either *G. duodenalis* assemblage A or B. It may therefore be alternative recognized routes of giardiasis transmission, such as through contamination of food or eating utensils (potentially with infectious lake water), during food preparation and/or consumption, through direct contact with infected individuals, or through unintentional consumption of lake water (e.g., when bathing), that is the primary mode of *G. duodenalis* infection within this study group.^1^^,^^4^

Interestingly, however, within this study group, there was a statistically significant positive association between single infection with *G. duodenalis* assemblage B and both self-reporting of abdominal (stomach) pain and self-reporting of loose stool (diarrhea), but no identified association between single infection with *G. duodenalis* assemblage A and any form of intestinal pathology. With regard to self-reported diarrhea specifically, these data are in agreement with some previous studies that have also found an association between single infection with *G. duodenalis* assemblage B and symptomatic diarrhea (but no such association between single infection with *G. duodenalis* assemblage A and diarrhea),^20^^,^^60^^,^^64^^–^^67^ yet are also in disagreement with other studies that have reached the opposite conclusion.^15^^,^^16^^,^^18^^,^^19^^,^^61^^,^^68^ This is also true for self-reported stomach pain; these data are in agreement with some previous studies that have also found an association between single infection with *G. duodenalis* assemblage B and stomach or abdominal pain (but no such association between single infection with *G. duodenalis* assemblage A and stomach/abdominal pain),^20^^,^^60^ but disagreement with other studies that have again reached the opposite conclusion.^15^^,^^19^^,^^64^^,^^68^

Currently, there is no clear correlation between *G. duodenalis* infection type and intestinal pathology.^69^ It has been suggested, however, that any discordance in either assemblage A or B being more (or less) associated with intestinal pathology is not a result of one or the other assemblage type being more pathogenic organisms, but rather that it is perhaps the least prevalent assemblage within a given locality that is responsible for causing intestinal pathology.^9^^,^^70^ This hypothesis, however, cannot be supported using these data because the prevalence of single infections with *G. duodenalis* assemblage A and B were similar. Alternatively, other studies have suggested that it is perhaps host–parasite factors, such as host age, nutritional status and immunological status, that are likely to play a major role in *G. duodenalis* assemblage-specific pathologies.^69^ Although this conclusion also cannot be reached using these data, this study does provide further evidence that more detailed future work investigating the potential drivers of any *G. duodenalis* assemblage-specific pathologies is warranted.

Additionally, although statistically insignificant (*P =* 0.06), there was a positive association between mixed infection with both *G. duodenalis* assemblages A and B and self-reporting of loose stool/diarrhea, as well as a statistically significant positive association between mixed infection with both assemblages and self-reporting of abdominal pain. Although an interesting observation, conclusions as to whether these associations are a result of individuals being infected with *G. duodenalis* assemblage B (found to be potentially more pathogenic within this study group), or whether there is some potential interplay between mixed infection with both assemblages and intestinal pathology, cannot be drawn using these data; thus, additional work is needed to scrutinize further whether infection with human-infecting *G. duodenalis* assemblages A and B has a more detrimental impact on intestinal health than single infection with just one assemblage.

Nevertheless, in keeping with previous studies, these data also suggest that there may be marked differences in the degree of pathology experienced by those infected with either *G. duodenalis* assemblages A and B, as well as between those burdened with single-assemblage (A or B) or mixed-assemblage (A and B) infections. Furthermore, these data also suggest that asymptomatic *G. duodenalis* infections may be linked to *G. duodenalis* infection type (potentially through single infection with a lesser pathogenic assemblage), as also suggested elsewhere.^70^

### Study limitations and future work.

An immediate limitation of this study is that *G. duodenalis* subassemblages AI–AIII, BIII, and BIV, which may also potentially differ in their pathogenic impact, could not be identified. To investigate any differences in pathological impact within *G. duodenalis* assemblage types, more thorough genetic analyses are needed.^71^ Additionally, to investigate and clarify further any potential associations between *G. duodenalis* infection status and intestinal pathology, additional and more extensive pathology testing, such as through measuring participant height and weight^5^ and through the use of ultrasonography to assess intestinal health,^4^^,^^72^^,^^73^ should be carried out.

Another limitation included the absence of additional genetic targets, such as the *gdh* locus, to confirm identified infection types—particularly in light of the fairly poor performance of the *G. duodenalis* assemblage-specific real-time PCR assay, targeting *tpi* loci, used here. Further development and optimization of reliable markers and assays for multi-locus *G. duodenalis* assemblage typing should continue to be carried out,^46^^,^^62^^,^^74^ ideally using real-time PCR to omit the need for time-consuming and expensive DNA sequencing and genetic analyses.

It should also be highlighted that previous studies have also found statistically significant differences in the severity of pathological symptoms, such as diarrhea, between those infected with either assemblage. As an interesting example, Homan and Mank^60^ reported an association between infection with *G. duodenalis* assemblage A and mild/intermittent diarrhea, whereas infection with *G. duodenalis* assemblage B was associated with severe/persistent diarrhea. Although severity of pathological symptoms such as diarrhea was not investigated here, future studies should seek to investigate further any associations between *G. duodenalis* infection type(s) and the severity of pathological symptoms, rather than just presence and absence of symptoms.

Furthermore, the pathogenic impact of other waterborne pathogens prevalent in this area known to cause similar intestinal pathologies, such as intestinal schistosomiasis and cryptosporidiosis, were not considered within any analyses and so may be confounding results, either through intestinal pathology being present within individuals not infected with *G. duodenalis* (but infected with other intestinal pathogens) or through intestinal pathology being exacerbated by coinfection with *G. duodenalis* and other intestinal pathogens.^9^ Future work should aim to evaluate and incorporate the pathogenic impact of coinfection with other intestinal pathogens to aid in clarifying the pathogenic impact of *G. duodenalis* assemblage types and infection status.

## CONCLUSIONS

Both *G. duodenalis* assemblage A and B single and mixed infections are common in school-age children situated along the southern shoreline of Lake Malawi, Mangochi district, Malawi, Within this study group, it was found that there may be marked differences in the degree of intestinal pathology experienced by those infected with *G. duodenalis* assemblage A compared with those infected with *G. duodenalis* assemblage B, as well as between those burdened with single- and mixed-assemblage infections. This study therefore highlights the importance of molecular methods that can be used to identify *G. duodenalis* assemblage types as well as the importance of investigating their impact on human pathology.

Molecular surveillance methods such as these should continue to be used, particularly in areas lacking adequate access to WASH infrastructure, to better understand the detrimental health impacts not only of infection with *G. duodenalis* but also of poor access to clean and safe drinking water and sanitation facilities. In doing so, not only can the pathological impact of human giardiasis be better understood, but, through improved disease surveillance and control and improved access to WASH infrastructure, a reduction in disease-associated pathologies experienced by the world’s most disadvantaged communities can also be achieved.

## Supplemental Materials


Supplemental materials

Supplemental materials
