# Supplementary material for: Molecular Epidemiology and Assemblage Typing of Giardia duodenalis in School-Age Children Situated along the Southern Shoreline of Lake Malawi, Malawi
Source: Am J Trop Med Hyg. 2023 Aug 7;109(3):626–39. doi: 10.4269/ajtmh.23-0156 (PMC10484258; doi:10.4269/ajtmh.23-0156)
Supplement: Supplementary file 1 [file tpmd230156.SD1.pdf]

Molecular epidemiology and assemblage typing of *Giardia duodenalis* in school-aged children situated along the southern shoreline of Lake Malawi, Malawi

Supplemental file 1.

Questionnaire October/November 2021

English

1. Do you go into the lake water?
2. If yes, is this (A) daily, (B) weekly, (C) monthly?
3. Do you swim in the lake water?
4. If yes, is this (A) daily, (B) weekly, (C) monthly?
5. Do you drink the lake water?
6. Have you ever received treatment for schistosomiasis (Bilharzia)?
7. If yes, when was the last time you received treatment?
8. Does it hurt when you urinate?
9. Does your urine have blood in it or is your urine red?
10. Do you currently have abdominal (stomach) pain?
11. Do you currently have loose stool or diarrhoea?
12. Have you ever lived anywhere else other than here?
13. Have you travelled to other areas of Malawi?
14. Do you live near any of the following livestock animals?

    (A) Cattle (cows), (B) Goats, (C) Sheep, (D) Other, (E) None
15. Do you have regular contact with any of the following livestock animals?

    (A)Cattle (cows), (B) Goats, (C) Sheep, (D) Other, (E) None

Chichewa

1. Kodi umapita ku nyanja?

2. Ngati inde, kangati? (A) Tsiku ndi tsiku, (B) Pa sabata / wiki, (C) Pa mwezi

3. Kodi umasambira mu nyanja?

4. Ngati inde, kangati? (A) Tsiku ndi tsiku, (B) Pa sabata / wiki, (C) Pa mwezi

5. Kodi umamwa madzi akunyanja?

6. Kodi unalandirapo mankhwala a likodzo?

7. Ngati inde, unalandira liti mankhwalawo?

8. Kodi ukumamva kupweteka pokodza?

9. Kodi mkodzo wako ukumakhala ndi magazi?

10. Kodi mmimba mwako mukupweteka?

11. Kodi ukupanga chimbuzi chofewa kapena ukutsegula mmimba?

12. Kodi unakhalapo ku malo ena kupatula komwe ukukhala pano?

13. Kodi udapita malo ena mu dziko la Malawi?

14. Ndi ziweto ziti zimene mumakhala nazo ku nyumba?

(A) Ng'ombe, (B) Mbuzi, (C) Nkhosa, (D) Zina - tchulani, (E) Sindikhala ndi ziweto

15. Ndi ziweto ziti zimene mumayandikana nazo?

(A) Ng'ombe, (B) Mbuzi, (C) Nkhosa, (D) Zina - tchulani, (E) Sindikumana ndi ziweto
