# Supplementary material for: Molecular Epidemiology and Assemblage Typing of Giardia duodenalis in School-Age Children Situated along the Southern Shoreline of Lake Malawi, Malawi
Source: Am J Trop Med Hyg. 2023 Aug 7;109(3):626–39. doi: 10.4269/ajtmh.23-0156 (PMC10484258; doi:10.4269/ajtmh.23-0156)
Supplement: Supplementary file 2 [file tpmd230156.SD2.pdf]

Molecular epidemiology and assemblage typing of *Giardia duodenalis* in school-aged children situated along the southern shoreline of Lake Malawi, Malawi

Supplemental file 2.

Table 1A: Primer and probe sequences used to detect and amplify a 62-bp *G. duodenalis*-specific rRNA region (18S gene) within the small subunit ribosomal ribonucleic acid (SSU RNA) and an 89-bp fragment of the PhHV-1 glycoprotein B gene[33]. Oligonucleotide sequences are shown from 5' end to 3' end.

	Name	Oligonucleotide sequence (5' – 3')
FW*	***Giardia*-80F**	GACGGCTCAGGACAACGGTT
RV†	***Giardia*-127R**	TTGCCAGCGGTGTCCG
Pro‡	***Giardia*-105T**	(**FAM¥**) CCCGCGGCGGTCCCTGCTAG
FW*	**PhHV-1-267s**	GGGCGAATCACAGATTGAATC
RV†	**PhHV-1-337as**	GCGGTTCCAAACGTACCAA
Pro‡	**PhHV-1-305tq**	(**Cy5¥**) TTTTTATGTGTCCGCCACCATCTGGATC

**Forward primer*
†Reverse primer
‡Probe
¥Fluorophore

Table 1B: Reaction mix used to carry out a duplex real-time PCR to detect and amplify a 62-bp *G. duodenalis*-specific rRNA region (18S gene) within the small subunit ribosomal ribonucleic acid (SSU RNA) and an 89-bp fragment of the PhHV-1 glycoprotein B gene. Adapted from[33].

	Concentration	Per 1 sample (µl)
ddH_2O	/	6.2
FW* (*Giardia*-80F)	100 nM	0.25
RV† (*Giardia*-127R)	100 nM	0.25
Pro‡ (*Giardia*-105T)	100 nM	0.25
FW* (PhHV-1-267s)	60 nM	0.15
RV† (PhHV-1-337as)	60 nM	0.15
Pro‡ (PhHV-1-305tq)	100 nM	0.15
AppPROBE No ROX mix (Appleton Woods, UK)	/	**12.5**
DNA	/	**5**
		**25**

**Forward primer*
†Reverse primer
‡Probe

Table 1C: Cycling conditions used to carry out a duplex real-time PCR to detect and amplify a 62-bp *G. duodenalis*-specific rRNA region (18S gene) within the small subunit ribosomal ribonucleic acid (SSU RNA) and an 89-bp fragment of the PhHV-1 glycoprotein B gene. Adapted from[33].

PCR step	Time	Temp	Cycles
Denaturation	15 min	95 ºC	/
Annealing	15 sec	95 ºC	45
	30 sec	60 ºC	

Table 2A: Primer sequences used to detect and amplify a 511-bp region of the *G. duodenalis* β-giardin (*bg*) locus for genotyping and phylogenetic analysis[34]. Both initial and nested PCR primer sequences are shown. Oligonucleotide sequences are shown from 5' end to 3' end.

	Name	Oligonucleotide sequence (5' – 3')	PCR
FW*	**G7**	AAGCCCGACGACCTCACCCGCAGTGC	Initial
RV†	**G759**	GAGGCCGCCCTGGATCTTCGAGACGAC	
FW*	**BGf**	GAACGAACGAGATCGAGGTCCG	Nested
RV†	**BGr**	CTCGACGAGCTTCGTGTT	

**Forward primer*
†*Reverse primer*

Table 2B: Reaction mix used to carry out a nested PCR to detect and amplify a 511-bp region of the *G. duodenalis* β-giardin (*bg*) locus for genotyping and phylogenetic analysis[34], with minor modifications. Both initial and nested PCR reaction mixes are shown.

	Concentration	x1 sample (µl) (Initial PCR)	Per 1 sample (µl) (Nested PCR)
ddH_2O	/	9.25	10.25
FW* (G7)	250 nM	0.625	/
RV† (G759)	250 nM	0.625	/
FW* (BGf)	250 nM	/	0.625
RV† (BGr)	250 nM	/	0.625
Hi-Fi Taq (New England Biolabs, UK)	/	12.5	12.5
DNA	/	**2**	**1** ***(of initial PCR product)***
		**25**	**25**

**Forward primer*
†*Reverse primer*

Table 2C: PCR conditions used to carry out a nested PCR to detect and amplify a 511-bp region of the *G. duodenalis* β-giardin (*bg*) locus for genotyping and phylogenetic analysis[34], with minor modifications. Both initial and nested PCR conditions are shown.

PCR step	Time	Temp	Cycles
Denaturation	30 sec	98 ºC	/
Annealing	10 sec	98 ºC	40
	30 sec	65 ºC (initial PCR)<br>64 ºC (nested PCR)	
	30 sec	72 ºC	
Extension	2 min	72 ºC	/

Table 3A: Primer and probe sequences used to detect and amplify *G. duodenalis* assemblage A- and B-specific fragments within the *tpi* locus (both 77-bp in length)[34,35]. Oligonucleotide sequences are shown from 5' end to 3' end.

	Name	Oligonucleotide sequence (5' – 3')	Target *G. duodenalis* assemblage
FW*	**GDAF**	CATTGCCCCTTCCGCC	A
RV†	**GDAR**	CTGCGCTGCTATCCTCAACTG	
Pro‡	**GDAT**	(**VIC**¥) CCATTGCGGCAAACA	
FW*	**GDBF**	GATGAACGCAAGGCCAATAA	B
RV†	**GDBR**	TCTTTGATTCTCCAATCTCCTTCTT	
Pro‡	**GDBT**	(**FAM**¥) AATATTGCTCAGCTCGAG	

**Forward primer*
†Reverse primer
‡Probe
¥ Fluorophore

Table 3B: Reaction mix used to carry out a duplex real-time PCR to detect and amplify *G. duodenalis* assemblage A- and B-specific fragments within the *tpi* locus (both 77-bp in length). Adapted from[34,35].

	Concentration	Per 1 sample (µl)
ddH$_2$O	/	3
FW* (GDAF): A	300 nM	0.75
RV† (GDAR): A	900 nM	2.25
Pro‡ (GDAT): A	100 nM	0.25
FW* (GDBF): B	300 nM	0.75
RV† (GDBR): B	900 nM	2.25
Pro‡ (GDBT): B	100 nM	0.25
AppPROBE No ROX mix (Appleton Woods, UK)	/	**12.5**
DNA	/	**3**
		**25**

**Forward primer*
†Reverse primer
‡Probe

Table 3C: Cycling conditions used to carry out a duplex real-time PCR to detect and amplify *G. duodenalis* assemblage A- and B-specific fragments within the *tpi* locus (both 77-bp in length). Adapted from[34,35].

**PCR step**	**Time**	**Temp**	**Cycles**
Denaturation	10 min	95 ºC	/
Annealing	15 sec	95 ºC	50
	60 sec	60 ºC	
